# Supplementary material for: The impact of Daylight Saving Time on dog activity
Source: PLoS One. 2025 Jan 29;20(1):e0317028. doi: 10.1371/journal.pone.0317028 (PMC11778716; doi:10.1371/journal.pone.0317028)
Supplement: S1 Table — Associated human ID is provided for companion dogs to note dyads. Information about breed, sex, age and weight is provided for canids. Age in years and weight in kilograms reflects that at time of data collection. Asterisks (*) next to ID notes individuals that were excluded for the study, see note below chart for more detailed reasoning. (DOCX) [file pone.0317028.s001.docx]

**S1 Table.** Summary of information for study participants. Associated human ID is provided for companion dogs to note dyads. Information about breed, sex, age and weight is provided for canids. Age in years and weight in kilograms reflects that at time of data collection. Asterisks (*) next to ID notes individuals that were excluded for the study, see note below chart for more detailed reasoning.

| **Associated human ID** | **Canid ID** | **Breed** | **Sex** | **Age (yrs)** | **Weight**  **(kg)** |
| --- | --- | --- | --- | --- | --- |
| Sled Dogs | | | | | |
| N/A | CAN001 | Alaskan husky | F | 8 | 22.22 |
| N/A | CAN002 | Alaskan husky | M | 2 | 24.94 |
| N/A | CAN003 | Alaskan husky | M | 2 | 22.68 |
| N/A | CAN004 | Alaskan husky | M | 2 | 20.41 |
| N/A | CAN005 | Alaskan husky | F | 2 | 18.14 |
| N/A | CAN006 | Alaskan husky | M | 3 | 27.21 |
| N/A | CAN007 | Alaskan husky | M | 3 | 29.48 |
| N/A | CAN008 | Alaskan husky | F | 7 | 22.68 |
| N/A | CAN009 | Alaskan husky | M | 4 | 31.75 |
| N/A | CAN010 | Alaskan husky | M | 11 | 29.48 |
| N/A | CAN011 | Alaskan husky | M | 5 | 31.75 |
| N/A | CAN012 | Alaskan husky | M | 9 | 27.21 |
| N/A | CAN013 | Alaskan husky | M | 5 | 27.21 |
| N/A | CAN014 | Alaskan husky | F | 8 | 23.13 |
| N/A | CAN015 | Alaskan husky | M | 7 | 27.21 |
| N/A | CAN016 | Alaskan husky | M | 4 | 22.68 |
| N/A | CAN017* | Alaskan husky | F | 4 | 27.21 |
| N/A | CAN018 | Alaskan husky | M | 2 | 25.85 |
| N/A | CAN019 | Alaskan husky | M | 4 | 28.57 |
| N/A | CAN020 | Alaskan husky | F | 6 | 23.58 |
| N/A | CAN021 | Alaskan husky | F | 2 | 17.69 |
| N/A | CAN022 | Alaskan husky | M | 4 | 27.21 |
| N/A | CAN023 | Alaskan husky | F | 7 | 27.21 |
| N/A | CAN024 | Alaskan husky | F | 6 | 22.68 |
| N/A | CAN025 | Alaskan husky | F | 6 | 22.68 |
| Companion Dogs | | | | | |
| CAH016* | CAN061* | Siberian Husky | F | 6 | 21.77 |
| CAH017* | CAN062* | Siberian Husky | F | 4 | 24.94 |
| CAH018 | CAN063 | Alaskan husky | M | 9 | 28.12 |
| CAH019 | CAN064 | Alaskan malamute/ Siberian husky (mix) | M | 2.8 | 38.10 |
| CAH020* | CAN065* | Husky | M | 3 | N/A |
| CAH020* | CAN066* | Siberian husky (mix) | M | 2 | 23.35 |
| CAH021 | CAN067 | Siberian husky | M | 9 | 27.21 |
| CAH021 | CAN068 | Alaskan Husky | M | 10 | 24.94 |
| CAH022 | CAN069 | Siberian Husky | M | 3 | 30.84 |
| CAH023 | CAN070 | Siberian Husky | F | 2 | 19.95 |
| CAH023 | CAN071 | Siberian Husky | F | 2 | 18.14 |
| CAH024* | CAN072* | Siberian Husky | M | 4 | 29.48 |
| CAH025 | CAN073 | Siberian Husky | M | 11 | 19.95 |
| CAH025 | CAN074 | Siberian Husky | F | 6 | 15.78 |
| CAH026 | CAN075 | Siberian Husky | M | 2 | 31.75 |
| CAH027 | CAN076 | Malamute/Alaskan husky mix | M | 4 | 37.64 |
| CAH028 | CAN077 | Alaskan Malamute | M | 2.5 | 61.24 |
| CAH028 | CAN078 | Siberian Husky | M | 8 | 24.94 |
| CAH029 | CAN079 | Siberian Husky | F | 8 | 24.94 |
| CAH030 | CAN080 | Malamute/Husky mix | M | 6 | 39.91 |
| CAH031 | CAN081 | Alaskan Malamute/Siberian Husky mix | F | 4.5 | 32.65 |
| CAH032* | CAN082* | Siberian Husky | M | 7 | 34.01 |
| CAH033 | CAN083 | Malamute | M | 6 | 44.45 |
| CAH033 | CAN084 | Malamute | F | 3 | 34.01 |
| CAH033 | CAN085 | Malamute | M | 5 | 72.57 |
| CAH033 | CAN086 | Alaskan Malamute/ Siberian Husky mix | F | 5 | 36.28 |
| CAH034* | CAN087* | Malamute/Husky mix | F | 6 | 31.75 |
| CAH035 | CAN088 | Siberian Husky | M | 5 | 31.75 |
| CAH035 | CAN089 | Siberian Husky | F | 3 | 16.78 |
| CAH035 | CAN090 | Siberian Husky | M | 3 | 27.21 |
| CAH035 | CAN091 | Siberian Husky | F | 7 | 20.41 |
| CAH036 | CAN092 | Siberian Husky | M | 2.5 | 29.02 |
| CAH036 | CAN093 | Siberian Husky | F | 3.5 | 24.94 |
| CAH036 | CAN094 | Siberian Husky | M | 8.5 | 33.11 |
| CAH037* | CAN095* | Siberian Husky | F | 2 | 20.41 |
| CAH038 | CAN096 | Siberian Husky | M | 3 | 24.94 |
| CAH039 | CAN097 | Siberian Husky | M | 3 | 25.85 |
| CAH040* | CAN098* | Siberian Husky/malamute mix | F | 2 | 24.94 |

*CAN017, CAN095, CAH037 were excluded due to missing sensor; CAN061, CAN062, CAN066, CAN082, CAN098, CAH016, CAH017, CAH020, CAH032 were excluded due to limited pretransition dates; CAN065, CAN072, CAH024, CAH040 were excluded due to incomplete data; CAN087 and CAH034 were excluded since caregiver did not make any changes to routines for DST.
