# Supplementary material for: The impact of Daylight Saving Time on dog activity
Source: PLoS One. 2025 Jan 29;20(1):e0317028. doi: 10.1371/journal.pone.0317028 (PMC11778716; doi:10.1371/journal.pone.0317028)
Supplement: S2 Table — (DOCX) [file pone.0317028.s002.docx]

**S2 Table.** Results from linear mixed-effects models on the effects of sex, age, and other dogs on morning onset activities for sled dogs and companion dogs.

| Sled Dogs | | | | |
| --- | --- | --- | --- | --- |
| Variable | Morning onset (handler) model  *N* = 239, *χ2* = 7.78, *p* = 0.020 | | Morning onset (sunrise) model  *N* = 239, *χ2* = 7.57, *p* = 0.023 | |
|  | *β* | *p* | *β* | *p* |
| Sex (ref: female) | 0.031 | 0.887 | 0.367 | 0.139 |
| Age | **-0.113** | **0.012** | -0.089 | 0.057 |
| Companion dogs | | | | |
| Variable | Morning onset (caregiver) model  *N* = 156, *χ2* = 3.74 *p* = 0.290 | | Morning onset (sunrise) model  *N* = 159, *χ2* = 4.25, *p* = 0.236 | |
|  | *β* | *p* | *β* | *p* |
| Sex (ref: female) | -0.186 | 0.526 | -0.620 | 0.142 |
| Age | -0.052 | 0.386 | -0.074 | 0.342 |
| Other dogs (ref: no) | 0.684 | 0.218 | -0.122 | 0.816 |

Significant effects (*p* < 0.05) are bolded.
