# Supplementary material for: The impact of Daylight Saving Time on dog activity
Source: PLoS One. 2025 Jan 29;20(1):e0317028. doi: 10.1371/journal.pone.0317028 (PMC11778716; doi:10.1371/journal.pone.0317028)
Supplement: S3 Table — (DOCX) [file pone.0317028.s003.docx]

**S3 Table.** Results from linear regressions on the effects of sex, age, and other dogs on morning onset activity difference between DST1 and Pre-DST for sled dogs and companion dogs.

| Sled Dogs | | | | |
| --- | --- | --- | --- | --- |
| Variable | Morning onset (handler) diff.  *N* = 24, *F* = 2.18, *p* = 0.138 | | Morning onset (sunrise) diff.  *N* = 24, *F* = NA, *p* = NA | |
|  | *β* | *p* | *β* | *p* |
| Sex (ref: female) | 4673.86 | 0.056 | - 2799.6 | 0.517 |
| Age | 10.58 | 0.9813 | 1069.5 | 0.207 |
| Companion dogs | | | | |
| Variable | Morning onset (caregiver) diff.  *N* = 29, *F* = 3.86, *p* = 0.021 | | Morning onset (sunrise) diff.  *N* = 29, *F* = 1.76, *p* = 0.180 | |
|  | *β* | *p* | *β* | *p* |
| Sex (ref: female) | -10963.4 | 0.058 | -0.620 | 0.142 |
| Age | **-2249.8** | **0.028** | -0.074 | 0.342 |
| Other dogs (ref: no) | -3254.4 | 0.593 | -0.122 | 0.816 |

Significant effects (*p* < 0.05) are bolded.
