# Supplementary figures and images for: The impact of Daylight Saving Time on dog activity
Source: PLoS One. 2025 Jan 29;20(1):e0317028. doi: 10.1371/journal.pone.0317028 (PMC11778716; doi:10.1371/journal.pone.0317028)

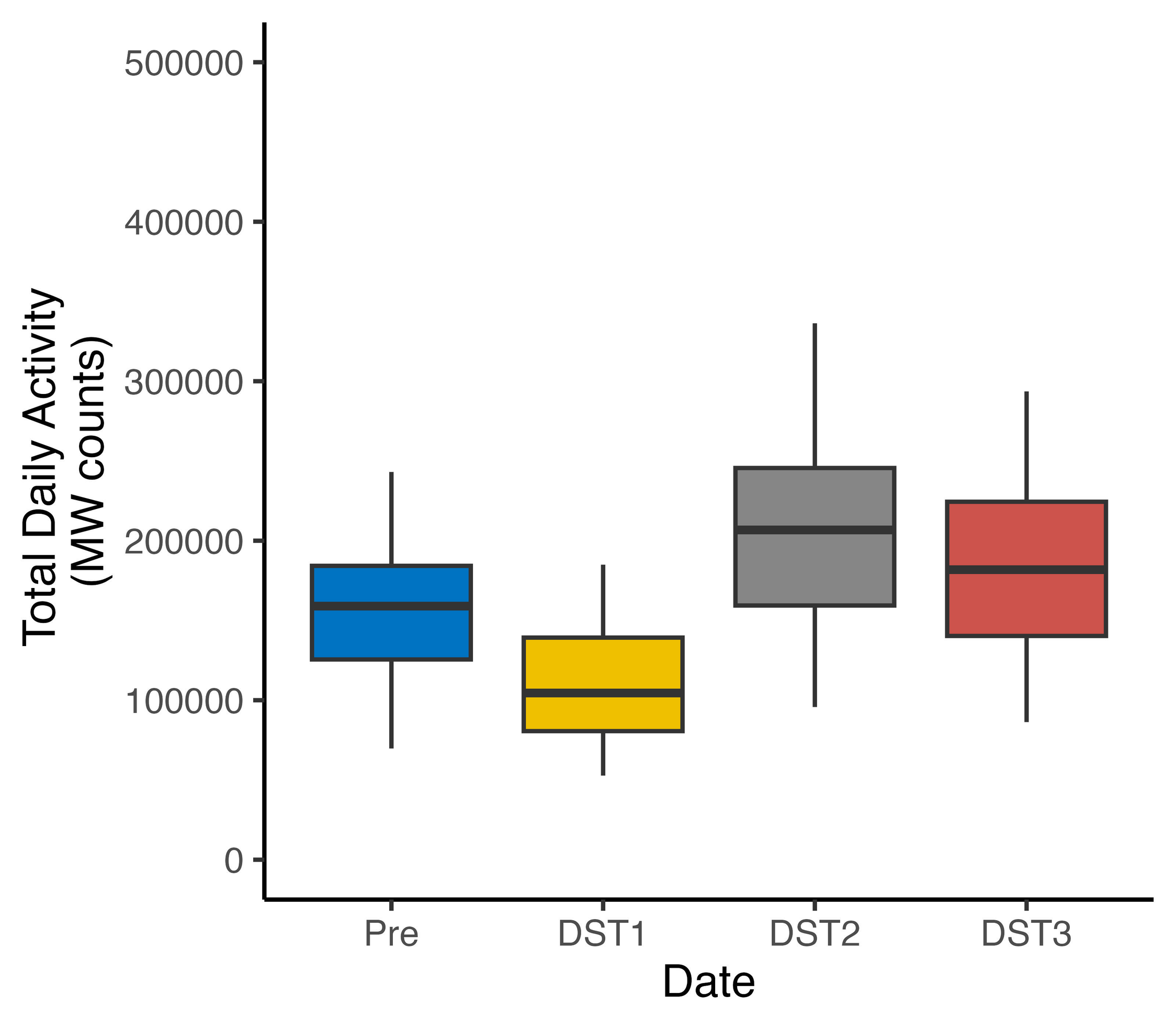

Supplement: S1 Fig — (TIF) [file pone.0317028.s004.tif]

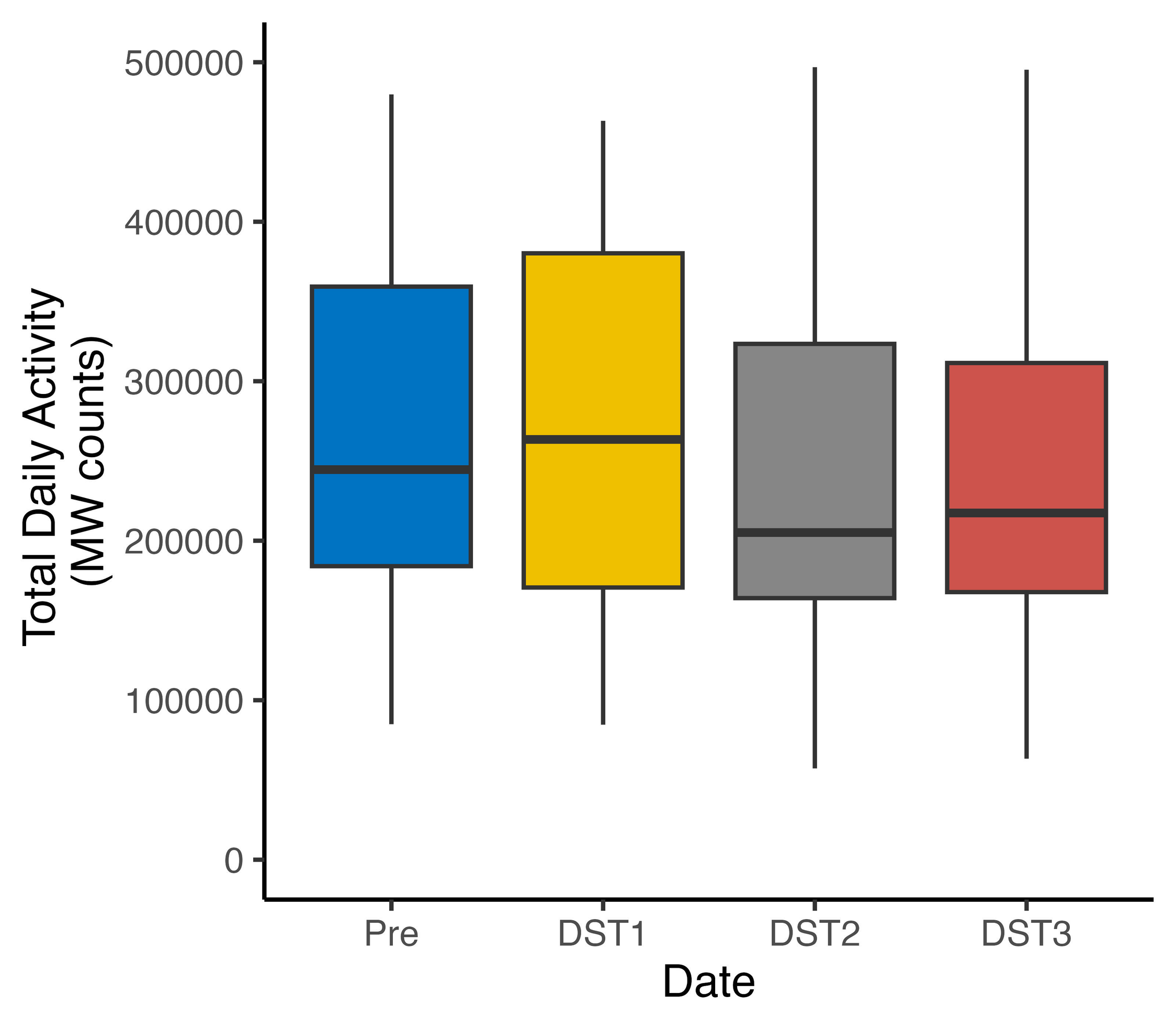

Supplement: S2 Fig — (TIF) [file pone.0317028.s005.tif]
