# Supplementary material for: The impact of Daylight Saving Time on dog activity
Source: PLoS One. 2025 Jan 29;20(1):e0317028. doi: 10.1371/journal.pone.0317028 (PMC11778716; doi:10.1371/journal.pone.0317028)
Supplement: S1 File — (DOCX) [file pone.0317028.s006.docx]

Supporting Information for “**The impact of Daylight Saving Time on dog activity”**

Lavania Nagendran, Ming Fei Li, David R. Samson, Lauren Schroeder

**Background Information for Sled Dogs**

Sled dogs at Haliburton Forest & Wild Life Reserve were housed in sex-specific outdoor enclosures (49.6 m by 12.2m). Within these enclosures up to three dogs shared kennels (averaging 1.5m x 2m). Dogs were given time during the day to roam free within the enclosures and the amount of time varied depending on environmental conditions. When handlers were not present on site, dogs were placed in their kennels (5 p.m. to 7:30 a.m.). Dogs were fed high-performance kibble once a day at 3 p.m. and the amount fed was based on weight. Training took place during the data collection period to prepare for peak visiting season and dogs were rotated for sledding practice runs.

**Total Daily Activity**

*Sled dogs*

We looked at total daily activity (sum of activity in the 24-hour period) for both sled dogs and companion dogs. To determine sled dogs’ activity measures pre-transition, we averaged activity measures across the seven days before DST transition (October 25 to October 31). We then compared the activity measure for each of the three consecutive days after DST transition to the pre-transition measure. In other words, we compared DST1 (November 1) to Pre, DST2 (November 2) to Pre, and DST3 (November 3) to Pre. Total daily activity was normally distributed so we used a paired t-test. We also used a one-way repeated measures ANOVA to see if total daily activity differed among the three post-DST days.

For total daily activity, we found that all three post-transition dates differed significantly from dogs’ pre-transition activity. Dogs were significantly less active in Post 1 (*p* < 0.001), more active in Post 2 (*p* < 0.001), and more active in Post 3 (*p* = 0.011) compared to pre-transition (S1 Fig.). The ANOVA found that activity was significantly different across the three post-transition dates (F = 54.24, *p* < 0.001). Post-hoc Wilcox pairwise comparisons (with Bonferroni corrections) showed that Post 1 was significantly less active than Post 2 (*p* < 0.001) and Post 3 (*p* < 0.001), and Post 2 was significantly more active than Post 3 (p = 0.026).

*Companion dogs*

To determine companion dogs’ activity measures pre-transition, we averaged activity measures across the days (range from 2 to 5) before DST transition (November 2 to November 6). We then compared the activity measure for each of the three consecutive days after DST transition to the pre-transition measure. In other words, we compared DST1 (November 7) to Pre, DST2 (November 8) to Pre, and DST3 (November 9) to Pre. Total daily activities were not normally distributed so we used Wilcoxan signed rank tests to compare DST 1, 2, and 3 to pre-transition activities, and we used the Friedman test to see whether there were differences among the three post-transition dates.

For total daily activity, we found that DST2 and DST3 differed from pre-transition activity, but not DST1 (*p* = 0.839; S2 Fig.). Dogs were significantly less active in DST2 (*p* < 0.001) and DST3 (*p* = 0.031) compared to pre-DST transition. The Friedman Test found that activity did not significantly differ across the three post-transition dates.

In companion dogs, lower levels of activity were observed on DST2 and DST3 compared to the pre-transition period. Companion dogs often showed a weekend effect where activity during weekends was higher than weekdays possibly a result of greater interactions with caregivers during their days off work [1]. The pre-transition period included both weekends and weekdays and since DST1 was a Sunday, higher levels of activity were expected. Sled dogs, on the other hand, had higher levels on DST2 and DST 3 compared to the pre-transition period. Our previous research found no weekend effect in Haliburton sled dogs during a separate data collection period (December 6, 2020 and January 19, 2021 [2]). This data was collected during the height of the COVID-19 pandemic where lockdown measures were strongly recommended by the provincial government. As a result, visitation was minimal during this period but sled dog training still took place. The higher levels of activity on the weekdays following DST may be due to greater training demands on these days compared to on weekends. Since the trends are opposite in our two study groups, largely based on human-mediated schedules, it does not appear that DST influenced the total daily activity of dogs.

**Effects of age, sex, and presence of other dogs on morning activity**

*Data analyses*

Here, we wanted to see whether dogs’ morning activities were affected by sex (male/female) and age (continuous) for sled dogs, and by sex, age, and presence of other dogs in the household (yes/no) for companion dogs.

First, we looked at morning onset activities for all dates (i.e., both pre- and post-DST transition dates). We built linear-mixed effects models (LMM) where the response variable was morning onset activity, fixed effects were the aforementioned variables. The response variable was positively skewed so to meet model assumptions, we applied a logarithmic transformation. To control for repeated observations, we included date and dog ID as random effects in the sled dog models. For the companion dog models, we set the random effects as dog ID nested within owner ID and date—we ended up removing date as an additional random effect from the companion dog morning onset (caregiver) model because the model had trouble converging due to limited number of observations. For overall model significance, we compared the full model with a null model containing only the random effects.

Second, we looked at the change in morning onset activities on DST1 compared to the averaged activities on Pre-DST dates (DST1 minus Pre-DST). In this set of analyses, we used linear regression where the response and dependent variable remained the same as the above analysis. Since the response variable included negative values (i.e., when activity on DST was less than Pre-DST), we did not apply a logarithmic transformation and used the raw activity counts instead. Again, we report the overall model significance by comparing the full model with a null mode.

Full model results are reported in Tables S2 (LMM) and S3 (linear regressions).

**References**

1. Woods HJ, Li MF, Patel UA, Lascelles BDX, Samson DR, Gruen ME. A functional linear modeling approach to sleep–wake cycles in dogs. Sci Rep. 2020;10: 22233. doi:10.1038/s41598-020-79274-2

2. Li MF, Nagendran L, Schroeder L, Samson DR. The activity patterns of nonworking and working sled dogs. Sci Rep. 2022;12: 7999. doi:10.1038/s41598-022-11635-5
