# Supplementary material for: The impact of Daylight Saving Time on dog activity
Source: PLoS One. 2025 Jan 29;20(1):e0317028. doi: 10.1371/journal.pone.0317028 (PMC11778716; doi:10.1371/journal.pone.0317028)
Supplement: Attachment — Submitted filename: Response to Reviewers.docx [file pone.0317028.s008.docx]

Response to Reviewers

We thank the editor and reviewer for their helpful comments. Please see our response to each comment in red text.

Journal Requirements:

When submitting your revision, we need you to address these additional requirements.

1. Please ensure that your manuscript meets PLOS ONE's style requirements, including those for file naming. The PLOS ONE style templates can be found at

<https://journals.plos.org/plosone/s/file?id=wjVg/PLOSOne_formatting_sample_main_body.pdf> and
<https://journals.plos.org/plosone/s/file?id=ba62/PLOSOne_formatting_sample_title_authors_affiliations.pdf>

We will rename all files according to PLOS ONE’s style requirements.

2. Thank you for stating the following financial disclosure:

“his research was supported through Discovery Grants from the Natural Sciences and Engineering Research Council of Canada (RGPIN-2020-04159 to L.S. and RGPIN-2020-05942 to D.R.S).”

Please state what role the funders took in the study. If the funders had no role, please state: "The funders had no role in study design, data collection and analysis, decision to publish, or preparation of the manuscript." If this statement is not correct you must amend it as needed.

Please include this amended Role of Funder statement in your cover letter; we will change the online submission form on your behalf.

We have added this amended Role of Funder statement at the bottom of our revision cover letter.

3. We note that you have indicated that there are restrictions to data sharing for this study. For studies involving human research participant data or other sensitive data, we encourage authors to share de-identified or anonymized data. However, when data cannot be publicly shared for ethical reasons, we allow authors to make their data sets available upon request. For information on unacceptable data access restrictions, please see <http://journals.plos.org/plosone/s/data-availability#loc-unacceptable-data-access-restrictions>.

Before we proceed with your manuscript, please address the following prompts:

a) If there are ethical or legal restrictions on sharing a de-identified data set, please explain them in detail (e.g., data contain potentially identifying or sensitive patient information, data are owned by a third-party organization, etc.) and who has imposed them (e.g., a Research Ethics Committee or Institutional Review Board, etc.). Please also provide contact information for a data access committee, ethics committee, or other institutional body to which data requests may be sent.

b) If there are no restrictions, please upload the minimal anonymized data set necessary to replicate your study findings to a stable, public repository and provide us with the relevant URLs, DOIs, or accession numbers. Please see <http://www.bmj.com/content/340/bmj.c181.long> for guidelines on how to de-identify and prepare clinical data for publication. For a list of recommended repositories, please see <https://journals.plos.org/plosone/s/recommended-repositories>. You also have the option of uploading the data as Supporting Information files, but we would recommend depositing data directly to a data repository if possible.

Please update your Data Availability statement in the submission form accordingly.

We decided to upload the raw data used to generate the results of our study as a “Supporting Information” file. We have updated our Data Availability statement in the submission form.

4. In this instance it seems there may be acceptable restrictions in place that prevent the public sharing of your minimal data. However, in line with our goal of ensuring long-term data availability to all interested researchers, PLOS’ Data Policy states that authors cannot be the sole named individuals responsible for ensuring data access (http://journals.plos.org/plosone/s/data-availability#loc-acceptable-data-sharing-methods).

Data requests to a non-author institutional point of contact, such as a data access or ethics committee, helps guarantee long term stability and availability of data. Providing interested researchers with a durable point of contact ensures data will be accessible even if an author changes email addresses, institutions, or becomes unavailable to answer requests.

Before we proceed with your manuscript, please also provide non-author contact information (phone/email/hyperlink) for a data access committee, ethics committee, or other institutional body to which data requests may be sent. If no institutional body is available to respond to requests for your minimal data, please consider if there any institutional representatives who did not collaborate in the study, and are not listed as authors on the manuscript, who would be able to hold the data and respond to external requests for data access? If so, please provide their contact information (i.e., email address). Please also provide details on how you will ensure persistent or long-term data storage and availability.

Data was uploaded as a Supporting Information file. Please see the above comment.

5. We note that there is identifying data in the Supporting Information file <Supporting Information.docx>. Due to the inclusion of these potentially identifying data, we have removed this file from your file inventory. Prior to sharing human research participant data, authors should consult with an ethics committee to ensure data are shared in accordance with participant consent and all applicable local laws.

Data sharing should never compromise participant privacy. It is therefore not appropriate to publicly share personally identifiable data on human research participants. The following are examples of data that should not be shared:

-Name, initials, physical address
-Ages more specific than whole numbers
-Internet protocol (IP) address
-Specific dates (birth dates, death dates, examination dates, etc.)
-Contact information such as phone number or email address
-Location data
-ID numbers that seem specific (long numbers, include initials, titled “Hospital ID”) rather than random (small numbers in numerical order)

Data that are not directly identifying may also be inappropriate to share, as in combination they can become identifying. For example, data collected from a small group of participants, vulnerable populations, or private groups should not be shared if they involve indirect identifiers (such as sex, ethnicity, location, etc.) that may risk the identification of study participants.

Additional guidance on preparing raw data for publication can be found in our Data Policy (<https://journals.plos.org/plosone/s/data-availability#loc-human-research-participant-data-and-other-sensitive-data>) and in the following article: <http://www.bmj.com/content/340/bmj.c181.long>.

Please remove or anonymize all personal information (location), ensure that the data shared are in accordance with participant consent, and re-upload a fully anonymized data set. Please note that spreadsheet columns with personal information must be removed and not hidden as all hidden columns will appear in the published file.

We have removed “Location” from the Supporting Information file.

6. Please review your reference list to ensure that it is complete and correct. If you have cited papers that have been retracted, please include the rationale for doing so in the manuscript text, or remove these references and replace them with relevant current references. Any changes to the reference list should be mentioned in the rebuttal letter that accompanies your revised manuscript. If you need to cite a retracted article, indicate the article’s retracted status in the References list and also include a citation and full reference for the retraction notice.

We have reviewed our reference list and ensured that it is complete and correct. We did not add or remove any references.

**Additional Editor Comments:**

In addition to the comments provided by the reviewer, the following points should be considered:

-The reasons for the exclusion of 8 dogs should be stated in the text and in more detail than in the supplementary material. In addition, possible effects of the exclusions should be discussed.

We included the reason for the exclusion of 1 sled dog and 8 companion dogs, and that we do not expect these exclusions to change our study findings. This information was added to the manuscript text (lines 105-115).

-The text should present information that can be highlighted from Table 1.

We have added text to the main manuscript that highlights pertinent information from Table 1 (lines 195-201).


[Note: HTML markup is below. Please do not edit.]

Reviewers' comments:

Reviewer's Responses to Questions

**Comments to the Author**

1. Is the manuscript technically sound, and do the data support the conclusions?

The manuscript must describe a technically sound piece of scientific research with data that supports the conclusions. Experiments must have been conducted rigorously, with appropriate controls, replication, and sample sizes. The conclusions must be drawn appropriately based on the data presented.

Reviewer #1: Yes

2. Has the statistical analysis been performed appropriately and rigorously?

Reviewer #1: Yes

3. Have the authors made all data underlying the findings in their manuscript fully available?

The [PLOS Data policy](https://can01.safelinks.protection.outlook.com/?url=http%3A%2F%2Ftrack.editorialmanager.com%2FCL0%2Fhttp%3A%252F%252Fwww.plosone.org%252Fstatic%252Fpolicies.action%2523sharing%2F1%2F010f01931b4c43d7-325846a0-4b59-4b90-85c6-f67d03aa0ddc-000000%2Fg2LyBhMZHCFfDx-HjLKwKd95Uuhi0KwLdFazdcWsPIU%3D184&data=05%7C02%7Clavania.nagendran%40mail.utoronto.ca%7Cb9537182d2cf4e9ba3b008dd02504b39%7C78aac2262f034b4d9037b46d56c55210%7C0%7C0%7C638669267607867226%7CUnknown%7CTWFpbGZsb3d8eyJFbXB0eU1hcGkiOnRydWUsIlYiOiIwLjAuMDAwMCIsIlAiOiJXaW4zMiIsIkFOIjoiTWFpbCIsIldUIjoyfQ%3D%3D%7C0%7C%7C%7C&sdata=2W58I0ZOe5ZK03tsJZjVuaZ%2F3D7RYGyYyTod6AS73h8%3D&reserved=0) requires authors to make all data underlying the findings described in their manuscript fully available without restriction, with rare exception (please refer to the Data Availability Statement in the manuscript PDF file). The data should be provided as part of the manuscript or its supporting information, or deposited to a public repository. For example, in addition to summary statistics, the data points behind means, medians and variance measures should be available. If there are restrictions on publicly sharing data—e.g. participant privacy or use of data from a third party—those must be specified.

Reviewer #1: Yes

4. Is the manuscript presented in an intelligible fashion and written in standard English?

PLOS ONE does not copyedit accepted manuscripts, so the language in submitted articles must be clear, correct, and unambiguous. Any typographical or grammatical errors should be corrected at revision, so please note any specific errors here.

Reviewer #1: Yes

5. Review Comments to the Author

Please use the space provided to explain your answers to the questions above. You may also include additional comments for the author, including concerns about dual publication, research ethics, or publication ethics. (Please upload your review as an attachment if it exceeds 20,000 characters)

Reviewer #1: The study addresses a relevant topic: the influence of time changes, such as Daylight Saving Time, on dogs' routines, which can have significant implications for the animals' well-being and the routine of their guardian. The research used accelerometers to measure the dogs' activity, providing objective and quantitative data on their behavior during the time transition. If data are available, I suggest (but not as a requirement) that association tests be carried out between the change in behavior and some variables, such as: Dogs' age: Including an analysis by age can be a valuable addition, since different age groups have different activity levels and behavior patterns. Presence of other animals in the guardian's home: This analysis would allow us to observe whether interaction with other animals influences canine behavior during the time change. Where the animal is raised (indoors or outdoors): Considering this variable may be relevant, since living conditions (whether the animal lives indoors or outdoors) can impact both activity patterns and the dogs' adaptation to time changes. The inclusion of these variables could provide a broader view of the factors that influence dogs' behavior when faced with time changes, increasing the value and practical applicability of the research results.

We thank the reviewer for these helpful suggestions. We agree that these additional analyses would provide further insight on dogs’ morning behavior and how they may respond to time changes. For sled dogs, we included additional analyses on how sex and age affect general morning activity levels and the change in morning activity on DST1 compared to Pre-DST. We did not include the presence of other dogs since all sled dogs live in kennels with other dogs and all kennels are located close to each other and are outdoors. For companion dogs, we included additional analyses on how sex, age, and presence of other dogs in the household affect general morning activity levels and the change in morning activity on DST1 compared to Pre-DST. We did not include location since all companion dogs, except for four, slept indoors during the study period. The four dogs that sometimes slept outside were all from the same household. Our main findings were that, compared to younger dogs, older sled dogs had lower morning onset activity around handler arrival time, and older companion dogs had lower morning onset activity based on caregiver got-up time on DST1 compared to Pre-DST.

We included the additional analyses and results in the Supporting Information file as well as in the main manuscript (lines 165-166, 183-184, 251-260). We also added an additional section to our Discussion interpreting results from these additional analyses (lines 336-348).

6. PLOS authors have the option to publish the peer review history of their article ([what does this mean?](https://can01.safelinks.protection.outlook.com/?url=https%3A%2F%2Ftrack.editorialmanager.com%2FCL0%2Fhttps%3A%252F%252Fjournals.plos.org%252Fplosone%252Fs%252Feditorial-and-peer-review-process%2523loc-peer-review-history%2F1%2F010f01931b4c43d7-325846a0-4b59-4b90-85c6-f67d03aa0ddc-000000%2F-BMDFtjwclcIsyi0bblB2ddgdE1_roiU6VyYBlxcS4I%3D184&data=05%7C02%7Clavania.nagendran%40mail.utoronto.ca%7Cb9537182d2cf4e9ba3b008dd02504b39%7C78aac2262f034b4d9037b46d56c55210%7C0%7C0%7C638669267607887478%7CUnknown%7CTWFpbGZsb3d8eyJFbXB0eU1hcGkiOnRydWUsIlYiOiIwLjAuMDAwMCIsIlAiOiJXaW4zMiIsIkFOIjoiTWFpbCIsIldUIjoyfQ%3D%3D%7C0%7C%7C%7C&sdata=F2Dotmu4VmBl4qjx%2B6Oz%2FBUsBXRzyfB2ItQwGKQ%2FMC0%3D&reserved=0)). If published, this will include your full peer review and any attached files.


If you choose “no”, your identity will remain anonymous but your review may still be made public.


**Do you want your identity to be public for this peer review?** For information about this choice, including consent withdrawal, please see our [Privacy Policy](https://can01.safelinks.protection.outlook.com/?url=https%3A%2F%2Ftrack.editorialmanager.com%2FCL0%2Fhttps%3A%252F%252Fwww.plos.org%252Fprivacy-policy%2F1%2F010f01931b4c43d7-325846a0-4b59-4b90-85c6-f67d03aa0ddc-000000%2F2lgjlPQvTk4EdA17_1rCaI2nZ-qzLuxA2lNDKpPxlqM%3D184&data=05%7C02%7Clavania.nagendran%40mail.utoronto.ca%7Cb9537182d2cf4e9ba3b008dd02504b39%7C78aac2262f034b4d9037b46d56c55210%7C0%7C0%7C638669267607907840%7CUnknown%7CTWFpbGZsb3d8eyJFbXB0eU1hcGkiOnRydWUsIlYiOiIwLjAuMDAwMCIsIlAiOiJXaW4zMiIsIkFOIjoiTWFpbCIsIldUIjoyfQ%3D%3D%7C0%7C%7C%7C&sdata=gkq2CukjTHk58yEgZ%2F9QQeNIx0nqunVV7vHFHYHFZH8%3D&reserved=0).

Reviewer #1: No
